# Randomized single-blinded study comparing sedation effectiveness and hemodynamic stability of remifentanil vs dexmedetomidine infusion for electrophysiology procedures in patients of National Heart Institute cathlab

**DOI:** 10.1007/s10840-024-01884-x

**Published:** 2024-08-07

**Authors:** Rozaini Hassan, Azlee Abdul Mutalib, Chen Yi Shang, Nirpal Singh Sachdev, Farkad Abdul Rahman, Esther Siew Lee Ling

**Affiliations:** Anaesthesia Department, National Heart Institute, 145, Jalan Tun Razak, 50400 Kuala Lumpur, Wilayah Persekutuan Kuala Lumpur Malaysia

**Keywords:** Remifentanil, Dexmedetomidine, Sedation effectiveness, Hemodynamic stability, Electrophysiology surgery

## Abstract

**Background:**

While studies comparing the effectiveness of remifentanil and dexmedetomidine are prevalent in other nations, using remifentanil alone is uncommon in Malaysia. This research aims to evaluate the effectiveness of sedation with remifentanil or dexmedetomidine infusion in monitored anesthesia care for electrophysiology procedures.

**Methods:**

This study is a single-center, single-blinded, prospective randomized clinical study. One hundred twenty patients were randomized into two groups (remifentanil vs dexmedetomidine). Demographic characteristics and clinical outcomes, including level of sedation, vital signs, and patient satisfaction were monitored and recorded.

**Results:**

Group R showed a higher mean observer’s assessment of alertness/sedation score (3.9 ± 0.7 vs 3.6 ± 0.8; *p* = 0.008), mean arterial pressure (92.0 ± 12.0 vs 83.0 ± 13.0 mmHg; *p* < 0.001), heart rate (82.0 ± 20.0 vs 73.0 ± 18.0 beats/min; *p* = 0.006), systolic blood pressure (139.0 ± 16.0 vs 123.0 ± 17.0 mmHg; *p* < 0.001) and diastolic blood pressure (75.0 ± 13.0 vs 69.0 ± 14.0 mmHg; *p* = 0.009) than Group D. Oxygen saturation (99.0 ± 1.0%; *p* = 0.220) and respiration rate (16.0 ± 3.0 breaths/min; *p* = 0.361) for both groups were the same. Adverse events, including hypotension, bradycardia, and respiratory depression were observed in both groups. Both groups gave positive responses ranging from fair to good for patient satisfaction.

**Conclusion:**

Dexmedetomidine is a better choice of anesthesia as it was associated with a higher level of sedation, more stable hemodynamics, lower incidence of adverse events, and better patient satisfaction.

**Graphical Abstract:**

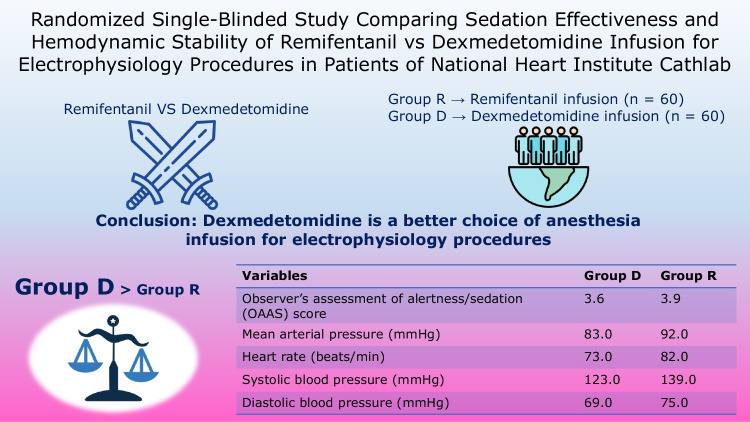

## Introduction

Pathway ablation, implantable internal cardioversion devices (ICDs), and pacemakers are among the prevalent and frequently performed procedures in electrophysiology (EP) labs. The engagement of anesthesiologists is due to the complexity of the procedures and patients’ poor cardiac status. It is crucial to maintain the patient’s cardiovascular stability during anesthesia in the EP procedures, as any hemodynamic changes may cause catastrophic consequences [1]. Therefore, prevention of myocardial ischemia in an already compromised cardiovascular system is vital. In the olden days, the dosage of opioids was high as used to suppress metabolic and surgical responses that can induce myocardial ischemia. However, high doses of opioids can cause chest wall rigidity, respiratory depression, and longer intensive care unit (ICU) and hospital stay [2].

The anesthesia community has developed a continuously improving range of methodologies for general and condition-specific sedation, increasing the decrease in the particular risk of pain and unease of patients undergoing a lot of procedures. The selection of ideal sedative regimens for minimum risk in patients undergoing electrophysiology studies and procedures has been a topic of debate in recent years, and numerous different regimens and experts exist. While there are studies evaluating the effectiveness of combining dexmedetomidine and remifentanil for sedation in electrophysiology procedures, there is a limited study comparing the two agents. The combination of dexmedetomidine and remifentanil achieved a deeper level of sedation, improved analgesic effects, and less respiratory depression during catheter ablation of atrial fibrillation compared with midazolam plus remifentanil, even at a lower dose of remifentanil [3]. Direct comparative studies are essential for providing robust evidence on the relative efficacy, safety, and patient outcomes associated with each sedative, allowing clinicians to make informed decisions regarding sedation management.

Remifentanil is a potent opioid characterized by rapid onset and offsets due to its metabolism by plasma esterase in blood and other tissues. Therefore, it is an organ-independent metabolism [4, 5]. Remifentanil acts on mu receptors and has few effects on delta and kappa receptors. It is 250 times more potent than morphine, which causes a profound blockade of sympathetic response to nociceptive stimulation [6]. With the increasing number of cardiac interventions, a rapid emergence agent is needed to shorten patient recovery time and hospital stay without compromising patient safety. Since remifentanil has an onset of action of 1 min and can rapidly achieve steady-state plasma levels with contact-sensitive half-time of 3–4 min, regardless of infusion duration, it has a better offer for cardiac surgery patients and is more cost-effective [7].

Dexmedetomidine is an α2-adrenoceptor agonist that has a sedative and analgesic effect. Since it causes minimal respiratory depression, it is widely used as sedation in cathlab, operating theatre, and ICUs [8, 9]. A study reported better control of fluctuation in mean arterial pressure (MAP) and heart rate (HR) with the administration of dexmedetomidine compared to control in patients with poor cardiac function undergoing laparoscopic cholecystectomy [10]. For individuals with healthy cardiac function, the administration of dexmedetomidine does not impair the biventricular systolic and diastolic function but only decreases cardiac output by reducing heart rate [11].

Both remifentanil and dexmedetomidine have their respective advantages when applied as an anesthetic agent during surgery in patients with poor cardiac function [7, 12, 13]. However, it is still largely unknown whether remifentanil or dexmedetomidine would be a better choice as an anesthetic agent when applied in the electrophysiology procedure. Therefore, this study aims to compare the effectiveness of sedation of remifentanil and dexmedetomidine infusion in monitored anesthesia care for patients with electrophysiology procedure from the aspects of sedation level and hemodynamic stability. In addition to providing insights on the comparison of effectiveness between remifentanil and dexmedetomidine, this study represents the effects of these two anesthetic agents in the context of the Malaysian population.

## Methods

### Study design

This was a single-center, single-blinded, prospective randomized clinical study for patients who underwent electrophysiology (EP) procedures. This study was approved by the Ethics Committee of the Malaysian National Heart Institute and written consent was obtained from participating patients. This study adhered to the applicable CONSORT guidelines.

Male and female patients aged older than 18 years administered to the institute for pacemaker implantation, internal cardioversion device (ICD) implantation and ablation of arrhythmia pathway procedures from January until June 2018 were included in the study. Those with upper airway obstruction and respiratory problems like chronic obstructive pulmonary disease (COPD), asthma, and lung tumor; body mass index (BMI) > 35; contraindications for sedation and inadequate fasting time were excluded as these high-risk patients were having a sedation with target control infusion (TCI).

After fasting, 120 patients were randomized into two groups on a ratio of 1:1 according to the type of anesthesia infusion received: (1) remifentanil (Group R; *n* = 60) and (2) dexmedetomidine (Group D; *n* = 60). Patients in Group R received 0.025–0.2 mcg kg^−1^ min^−1^ infusion of remifentanil, while Group D patients received 0.2–0.7 mcg kg^−1^ h^−1^ continuous infusion of dexmedetomidine.

### Procedures

All patients were administered with at least 20G intravenous branula and oxygen delivery via face mask at 3 L/min. Then, they were applied with standard monitoring of electrocardiogram (ECG), non-invasive blood pressure (NIBP) measurement, oxygen saturation (SPO2), and end-tidal carbon dioxide gradient (ETCO2). The level of sedation was maintained around 3–4 on the observer’s assessment of alertness/sedation (OAAS) score; 0 = does not respond to deep stimuli, 1 = does not respond to mild prodding or shaking, 2 = respond only after mild prodding or shaking, 3 = respond only after name is called loudly or repeatedly, 4 = lethargy response to name spoken in normal tone, 5 = respond readily to name spoken in normal tone.

An anesthesiologist trainee was presented throughout the procedure to monitor the patient as per hospital protocol. Patients randomized to the remifentanil group were infused with remifentanil at a rate of 0.025 to 0.2 mcg kg^−1^ min^−1^. The remifentanil will increase or decrease by 0.05 mcg kg^−1^ min^−1^ to achieve OAAS of 3 to 4, maintain SPO2 > 95% and blood pressure (BP), heart rate (HR), mean arterial pressure (MAP) ± 20% within the baseline. The patients in the dexmedetomidine group had an infusion rate of 0.2 to 0.7 mcg kg^−1^ h^−1^ for dexmedetomidine, which then was increased or decreased by 0.1 mcg kg^−1^ h^−1^ to achieve the same OAAS of 3 to 4, maintain SPO2 > 95% and BP, HR, MAP ± 20% within baseline.

**Fig. 1:**
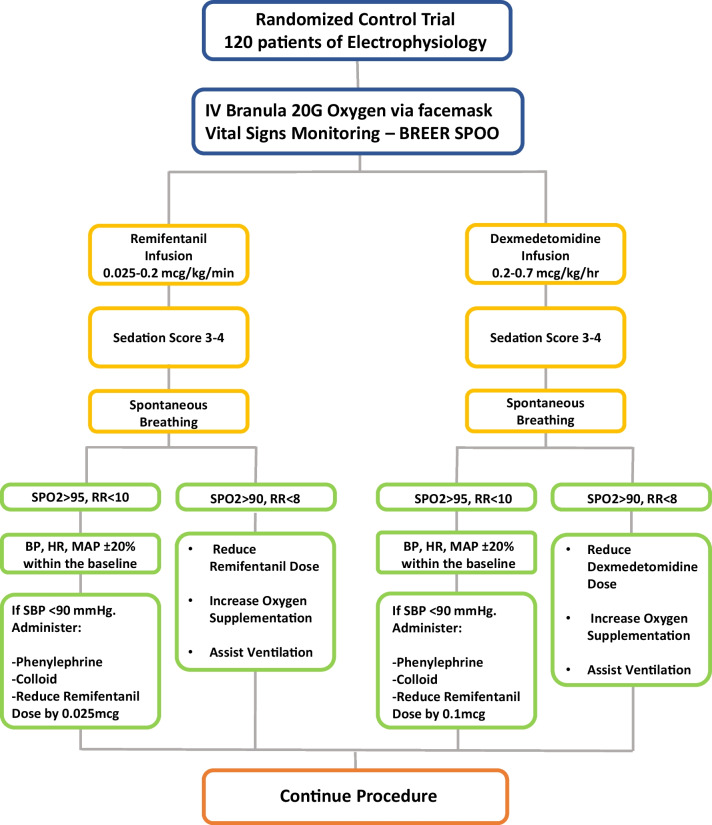
Flowchart of anesthesia administration procedures. BP, blood pressure; HR, heart rate; SPO2, oxygen saturation; RR, respiration rate; MAP, mean arterial pressure; SBP, systolic blood pressure

Bradycardia (HR < 50/min) was treated with intravenous atropine (Pfizer Inc., USA), except in the case of heart block. The oxygen supplement was increased to 5 L/min when SPO2 < 94% and respiration rate (RR) < 8. The concentration of remifentanil either decreased or discontinued the infusion if the patient did not respond to these treatments. On the contrary, the concentration of remifentanil was increased by 0.05 mcg kg^−1^ min^−1^ during cases of hypertension (BP > 20% baseline), HR > 90/min, OAAS score > 4, and pain score > 4. A similar procedure was applied to Group D with the replacement of remifentanil for dexmedetomidine (Pfizer Inc., USA). The flowchart of the entire procedure is shown in Fig. 1.

### Data collection

Demographic characteristics of all patients were recorded, including age, gender, ethnicity, BMI, New York Heart Association (NYHA) classification, ejection fraction (EF) comorbidity, and types of surgical procedures. Clinical outcomes were monitored and recorded at baseline and throughout the procedure, including OAAS and vital signs assessment (MAP, HR, SBP, DBP, SPO2, and RR). Adverse events related to anesthesia administration, such as hypotension, bradycardia, respiratory depression, and postoperative nausea and vomiting (PONV) were observed and recorded during the procedure. In addition, patient satisfaction with anesthesia was assessed during recovery.

### Statistical analysis

Categorical data were expressed in number (*n*) and percentage (%), while continuous data were presented in mean ± standard deviation (SD) and median. All data were analyzed with the IBM SPSS Statistics Software Version 24 (SPSS Inc., USA). The mean of continuous data between groups was analyzed and compared using a paired *t*-test, whereas categorical data were analyzed using a Chi-square test. The difference in mean with a *p*-value < 0.05 was considered significant.

## Results

**Fig. 2:**
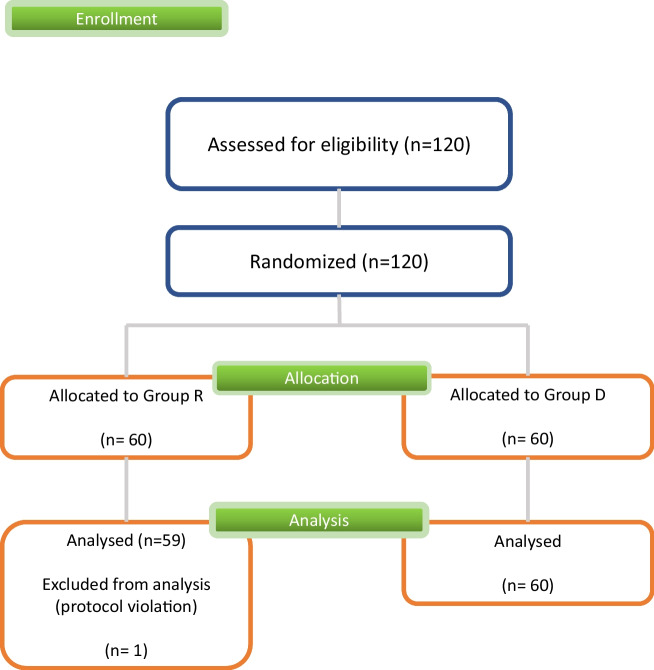
CONSORT flowchart describing the study

A total of 120 patients were recruited in this study, with 60 patients randomized into Group R and Group D, respectively. However, one patient from Group R was excluded from the analysis due to the violation of the surgical procedure (Fig. 2).

The mean age of patients in Group R (57.4 ± 14.9 years) was slightly younger than the mean age of Group D patients (59.6 ± 16.8). There were more male than female patients in both groups. However, the differences in these variables between the two groups were not significant (*p* > 0.05). Other variables that were not significant between the two groups included ethnicity, BMI, EF, and type of procedures. Meanwhile, significant differences were observed between the two groups in terms of NYHA (*p* = 0.032) and diabetes mellitus (*p* = 0.013). The number of patients with type II and III heart failure, according to NYHA, was lower in Group R compared to Group D. More patients from Group D had diabetes mellitus compared to Group R. The details of all patients are presented in Table 1.

**Table 1:** Demographic characteristics of patients

Variables	Group R (*n* = 59)	Group D (*n* = 60)	*p*-value
*Age (years)*			
Mean ± SD	57.4 ± 14.9	59.6 ± 16.8	0.258
Median (Q1, Q3)	62.0 (48.0, 68.0)	63.5 (48.0, 71.0)	
*Gender*			0.296
Male	38 (64.4%)	33 (55.0%)	
Female	21 (35.6%)	27 (45.0%)	
*Ethnicity*			0.926
Malay	33 (55.9%)	35 (58.3%)	
Chinese	14 (23.7%)	13 (21.7%)	
Indian	11 (18.6%)	10 (16.7%)	
Others	1 (1.7%)	2 (3.3%)	
*BMI (kg/m*^*2*^*)*			
Mean ± SD	26.4 ± 4.3	26.4 ± 4.7	0.834
Median (Q1, Q3)	26.0 (23.0, 30.0)	27.0 (24.3, 29.0)	
*NYHA classification*			0.032*
I	12 (24.0%)	5 (8.8%)	
II	24 (48.0%)	28 (49.1%)	
III	14 (28.0%)	24 (42.1%)	
*Ejection fraction*			0.915
< 35 (poor)	12 (22.2%)	15 (25.4%)	
35–49 (impaired)	12 (22.2%)	12 (20.3%)	
> 50 (good)	30 (55.6%)	32 (54.2%)	
*Comorbidities*			
Hypertension	31 (52.5%)	34 (56.7%)	0.651
Diabetes mellitus	15 (25.4%)	28 (47.5%)	0.013*
*Types of surgery*			
PPM implantation	10 (17.5%)	15 (25.9%)	0.281
ICD implantation	4 (7.0%)	10 (17.2%)	0.094
Box change	3 (5.3%)	5 (8.6%)	0.479
RFA	11 (19.3%)	6 (10.3%)	0.178
CRT-D	8 (14.0%)	5 (8.6%)	0.359
CRT-P	4 (7.0%)	3 (5.2%)	0.679
Atrial fibrillation ablation (cryoablation)	10 (17.5%)	6 (10.3%)	0.265
CARTO ablation	1 (1.8%)	4 (6.9%)	0.176
Others	6 (10.5%)	4 (6.9%)	0.490

Abbreviations: *CARTO* cardiac arrhythmias catheter, *CRT-D* Cardiac resynchronization therapy defibrillator, *CRT-P* cardiac resynchronization therapy pacemaker, *ICD* internal cardioversion device, *NYHA* New York Heart Association, *PPM* permanent pacemaker, *RFA* radiofrequency ablation, *SD* standard deviation; *p < 0.05, differences were significant

The clinical outcomes of all patients are presented in Table 2. As for the sedation assessment, patients from Group R (3.9 ± 0.7) showed significantly higher mean OAAS scores than patients from Group D (3.6 ± 0.8; *p* = 0.008).

**Table 2:** Clinical outcomes of patients

Variables	Group R (*n* = 59)	Group D (*n* = 60)	*p*-value
*Observer’s assessment of alertness/sedation (OAAS) score*			
Mean ± SD	3.9 ± 0.7	3.6 ± 0.8	0.008*
Median (Q1, Q3)	4 (4, 4)	4 (3, 4)	
Range score 1–2	1 (1.8%)	2 (3.6%)	0.558
Range score 3–5	55 (98.2%)	54 (96.4%)	
*Pain score*			
Mean ± SD	0.8 ± 1.0	0.5 ± 0.9	0.061
Median (Q1, Q3)	0.0 (0.0, 1.5)	0.0 (0.0, 1.0)	
Range score < 3	56 (98.2%)	55 (100.0%)	0.324
Range score > 4	1 (1.8%)	0 (0.0%)	
*Vital signs*	Mean ± SD	Mean ± SD	
Mean arterial pressure (MAP) (mmHg)	92.0 ± 12.0	83.0 ± 13.0	< 0.001*
Heart rate (HR) (beats/min)	82.0 ± 20.0	73.0 ± 18.0	0.006*
Systolic blood pressure (SBP) (mmHg)	139.0 ± 16.0	123.0 ± 17.0	< 0.001*
Diastolic blood pressure (DBP) (mmHg)	75.0 ± 13.0	69.0 ± 14.0	0.009*
Oxygen saturation (SPO_2_) (%)	99.0 ± 1.0	99.0 ± 1.0	0.220
Respiration rate (RR) (breaths/min)	16.0 ± 3.0	16.0 ± 3.0	0.361
*Adverse events*			
Hypotension	5 (8.5%)	10 (16.7%)	0.263
Bradycardia	6 (10.2%)	1 (1.7%)	0.126
Respiratory depression (SPO_2_ < 94%)	16 (27.1%)	2 (3.3%)	< 0.001*
*Patient satisfaction*			0.205
Fair	7 (11.9%)	2 (3.3%)	
Good	38 (64.4%)	41 (68.3%)	

*p < 0.05, differences were significant

In terms of vital signs assessment, significant differences in MAP (*p* < 0.001), HR (*p* = 0.006), SBP (*p* < 0.001), and DBP (*p* = 0.009) were observed between the two groups. Generally, patients in Group R recorded higher values of MAP, HR, SBP, and DBP compared to Group D patients. On the other hand, patients from both groups recorded the same values as observed in SPO2 and RR.

**Fig. 3:**
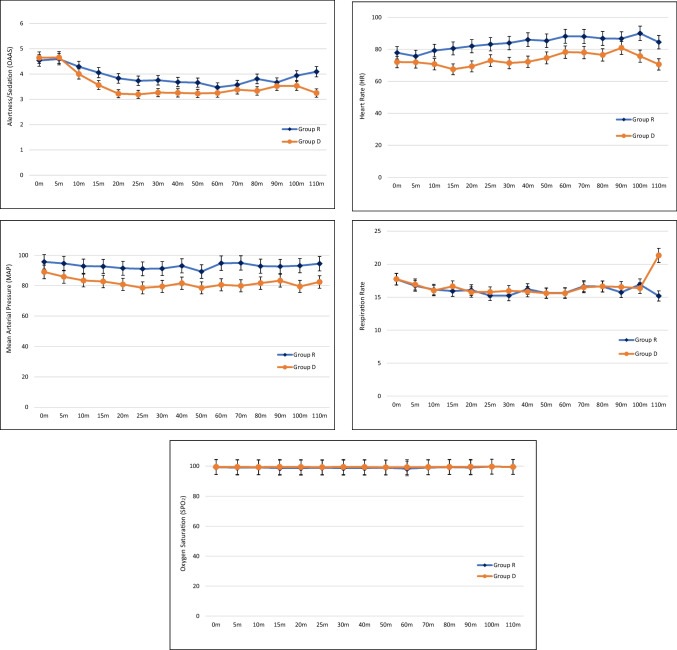
Comparison of hemodynamic stability between Group R and Group D for the duration of 0–110 min of surgery

In terms of adverse events occurrence, complications such as hypotension, bradycardia and respiratory depression (SPO2 < 94%) were observed in patients. The number of bradycardia (*n* = 6; 10.2% vs *n* = 1; 1.7%) and respiratory depression (*n* = 16; 27.1% vs *n* = 2; 3.3%) cases were higher in Group R compared to Group D. The difference between groups for respiratory depression occurrence was significant (*p* < 0.001), while the difference for bradycardia occurrence was not significant (*p* = 0.126). However, the number of hypotension cases in Group R (*n* = 5; 8.5%) was lower than in Group D (*n* = 10; 16.7%), but the difference was not significant (*p* = 0.263). In terms of patient satisfaction, all patients provided positive responses ranging from fair to good for both remifentanil and dexmedetomidine during surgery. The use of dexmedetomidine received slightly more “good" responses from patients than remifentanil, although the difference was not significant. As shown in Fig. 3, changes in all parameters for both Group R and Group D remained stable throughout the surgery.

## Discussion

Results of the study show a significantly lower mean score of OAAS in Group D than in Group R, indicating a higher level of sedation in patients administered with dexmedetomidine compared to patients administered with remifentanil. This finding is consistent with that reported by previous literature. For example, a study that used bispectral index (BIS) values to assess the depth of sedation during transesophageal echocardiography (TEE) reported patients who received dexmedetomidine had lower BIS values (70–80) than those who received propofol (80–85). Their findings suggested dexmedetomidine administration was superior as it induced deeper sedation than propofol. Although there is no comparison between dexmedetomidine and remifentanil in terms of the depth of sedation was made, the study proved that dexmedetomidine can reduce the nerve sensitivity of patients to pain and enhance the anesthetic effects during electrophysiology surgery [14].

As for the hemodynamic assessment, minimal fluctuations were observed in MAP, HR, SBP, DBP, SPO2, and RR for both Group R and Group D from 0 to 110 min during the surgery. This indicates that the administration of both remifentanil and dexmedetomidine during electrophysiology surgery is safe as both drugs can maintain adequately stable hemodynamics in patients with reduced cardiac reserve. According to the results, the measurements of MAP, HR, SBP, and DBP for Group D were significantly lower than those in Group R, thus consistent with findings reported by previous studies [7, 12]. This is because dexmedetomidine has high affinity and selectivity to the α2-adrenoceptors. Through binding and activation of the presynaptic α2-adrenoceptors in the central nervous system, dexmedetomidine induces a sympatholytic effect, which lowers the MAP and HR. Through the activation of α2-adrenoceptors in the endothelial cells, dexmedetomidine induces vasodilation, which lowers the SBP and DBP [15]. The superiority of dexmedetomidine in maintaining stable hemodynamics over remifentanil has been proven in previous literature. A cohort study reported lower mean values of MAP (91 ± 14 vs 91 ± 19 mmHg; *p* = 0.879), HR (99 ± 15 vs 104 ± 20 beats/min; *p* = 0.192), SBP (140 ± 23 vs 143 ± 29 mmHg; *p* = 0.548), and DBP (65 ± 17 vs 66 ± 14 mmHg; *p* = 0.746) for dexmedetomidine group than remifentanil group over the course of study (1–72 h) during cardiac surgery among patients with non-invasive ventilation intolerance [7]. Besides, fewer fluctuations were observed in the readings for the dexmedetomidine group than those in the remifentanil group [12]. This indicates the effectiveness of dexmedetomidine in maintaining a stable hemodynamic profile in patients undergoing electrophysiology procedures.

Meanwhile, the mean values of SPO2 and RR for both experimental groups were the same, which contradicts previous findings, where lower mean values of SPO2 (97% vs 98%; *p* = 0.368) and RR (25 ± 8 vs 28 ± 8; *p* = 0.157) were observed in the dexmedetomidine group than those in the remifentanil group. Hence, this suggests dexmedetomidine exerts a better effect in reducing respiratory drive than remifentanil [7]. Nonetheless, the readings of SPO2 and RR remained stable with minimal fluctuation throughout the surgery for Group R and Group D in this study without any severe case of respiratory depression, which proves the suitability of using remifentanil and dexmedetomidine as anesthesia for electrophysiology surgery in patients with poor left ventricular function.

In terms of adverse events occurrence, the incidence of bradycardia and respiratory depression were greater among Group R compared to Group D. In contrast, the occurrence of hypotension was greater among Group D than Group R. The result regarding the occurrence of hypotension was consistent with the findings described in the previous report, which showed a higher incidence of hypotension following the administration of dexmedetomidine compared to remifentanil [16]. This is due to the pharmacodynamic properties of dexmedetomidine, which induces the reduction of BP during surgery that eventually leads to hypotension [15]. Similarly, reflection on the occurrence of respiratory depression in this study is also in line with previous reports, which described minor respiratory depression for dexmedetomidine usage but a higher incidence of respiratory depression for remifentanil [15]. As for the occurrence of bradycardia, unlike the current study, the previous report has not associated the adverse event with remifentanil administration, but its occurrence is often associated with dexmedetomidine administration. A review that analyzed results from six studies involving dexmedetomidine usage found that dexmedetomidine significantly increases the risk of bradycardia (RR = 2.78; 95% CI = 2.00, 3.78; I2 = 0%) [16]. Another publication attributes the association of dexmedetomidine to bradycardia as a consequence of pre- and postsynaptic α2-adrenoceptor activation that leads to hemodynamic alterations and reflex bradycardia [15]. Despite the occurrence of these adverse events, their effects were mild and did not cause any fatal consequences to the patients.

In terms of patient satisfaction, all patients provided positive responses regarding the administration of remifentanil and dexmedetomidine, with slightly better satisfaction for dexmedetomidine. Consistent with current results, a previous study also reported better patient and anesthesiologist satisfaction with dexmedetomidine during the TEE procedures [14]. The use of dexmedetomidine was associated with more stable hemodynamics, lesser respiratory depression, lesser intraoperative bleeding, and reduced recovery time in comparison with remifentanil [12].

There are several limitations in this study. Parameters such as previous respiratory disease, dementia, and the amount of intraoperative bleeding and recovery time were not recorded and analyzed. The inclusion of these parameters can determine whether the administration of remifentanil and dexmedetomidine has any influence on these factors. Moreover, a follow-up assessment such as delirium and gastrointestinal complication was not performed on the patients. Without this, the occurrence of any postoperative complications, possibly associated with anesthesia administration, could not be detected. Besides, since this is a single-center study focusing on patients recruited from a single heart institute, the results obtained do not represent the overall anesthetic effects of remifentanil and dexmedetomidine on the entire Malaysian population. Besides, this study excluded high-risk patients, such as those with upper airway obstruction, respiratory problems like COPD, asthma, lung tumor, and patients with a BMI greater than 35, which consequently limit the generalizability of our findings to these excluded populations. Also, another study is required to investigate the suitability of remifentanil as a single agent in poor left ventricle function. Therefore, further research, with a larger sample size encompassing patients recruited from multiple centers, a longer follow-up period, and the inclusion of more extensive parameters such as follow-up assessment and age-related analyses on the sedation outcomes, should be conducted in the future to give a better overview of the anesthetic effects of remifentanil and dexmedetomidine.

## Conclusion

Dexmedetomidine is associated with higher levels of sedation, less pain, more stable hemodynamics, according to lower MAP, HR, SBP, and DBP, lower incidence of adverse events, and better patient satisfaction than remifentanil. Therefore, dexmedetomidine is a better choice of sedation in electrophysiology procedures.
